# Chromosome-level genome assembly of the jumping spider *Spartaeus platnicki*

**DOI:** 10.1038/s41597-026-07685-3

**Published:** 2026-06-24

**Authors:** Zhiyong Yang, Aidie Chen, Feng Zhang, Junxia Zhang

**Affiliations:** 1 Key Laboratory of Zoological Systematics and Application of Hebei Province, College of Life Sciences, Hebei University, Baoding, Hebei 071002 P. R. China; 2 Hebei Basic Science Center for Biotic Interaction, Hebei University, Baoding, Hebei 071002 P. R. China; 3 Engineering Research Center of Ecological Safety and Conservation in Beijing-Tianjin-Hebei (Xiong’an New Area) of MOE, Baoding, Hebei 071002 P. R. China

## Abstract

Jumping spiders, a diverse and charismatic group of invertebrates, are renowned for their highly specialized visual systems, complex courtship behaviours, and broad ecological adaptability. However, the persistent lack of high-quality genomic resources has hindered investigations into the genetic basis and phenotypic evolution of these key traits. To address this gap, we performed a *de novo* assembly of a chromosome-level genome for *Spartaeus platnicki*, a species representing the basal lineages of jumping spiders. This assembly, achieved by integrating PacBio HiFi long reads, Illumina short reads, RNA-seq, and Hi-C data, comprises 15 pseudo-chromosomes including the X1 and X2 sex chromosomes, spanning a total length of 3.71 Gb with a scaffold N50 of 262 Mb and a BUSCO completeness of 98.60%. Repetitive elements account for approximately 65.57% of the genome. We annotated 15,660 protein-coding genes, achieving a BUSCO completeness of 97.60%. This high-quality genome establishes a foundational resource for investigating the genetic architecture underlying key traits and their phenotypic evolution in jumping spiders.

## Background & Summary

Spiders represent one of the most diverse groups of terrestrial predators, with over 53,000 known species^1^. Among them, jumping spiders (Salticidae) constitute the most species-rich lineage, encompassing nearly 7,000 described species^1^. They have successfully colonized most terrestrial environments worldwide, and are particularly noted for their high spatial acuity vision^2^. Current phylogenetic frameworks divide Salticidae into seven subfamilies: the highly diverse Salticinae, which accounts for over 90% of the family’s species diversity, and six other subfamilies collectively referred to as “basal lineages” or “non-salticines”, representing less than 10% of the diversity^3^. While the Salticinae are crucial for genomic investigations, obtaining high-quality genomic data from the basal lineages is also essential for a comprehensive understanding of the family’s evolutionary history.

**Fig. 1:**
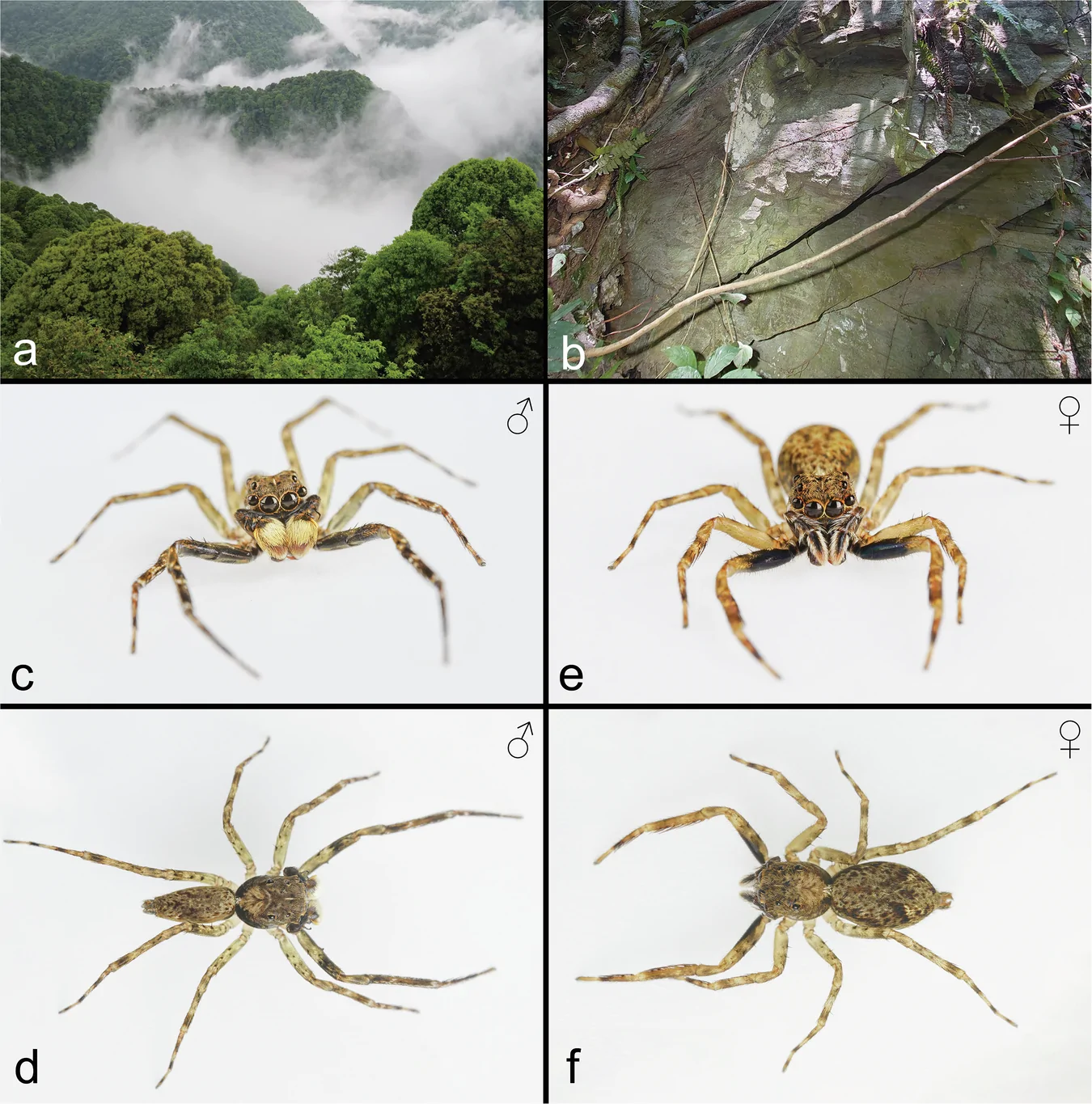
Habitats and live specimens of *Spartaeus platnicki*.

We here focus on *Spartaeus platnicki*, a species commonly found in rocky crevices of southern China’s montane forests (Fig. 1). As a representative of the basal subfamily Spartaeinae, *S. platnicki* exhibits distinct traits compared to Salticinae, such as the notably larger posterior median eyes^3^, and the construction of moderate-sized silky sheets that often have a role in prey capture—a behavior uncommon in most salticids, which typically do not build webs for predation^4,5^. Thus, *S. platnicki* serves as a critical model for understanding morphological and behavioral evolution in jumping spiders.

**Fig. 2:**
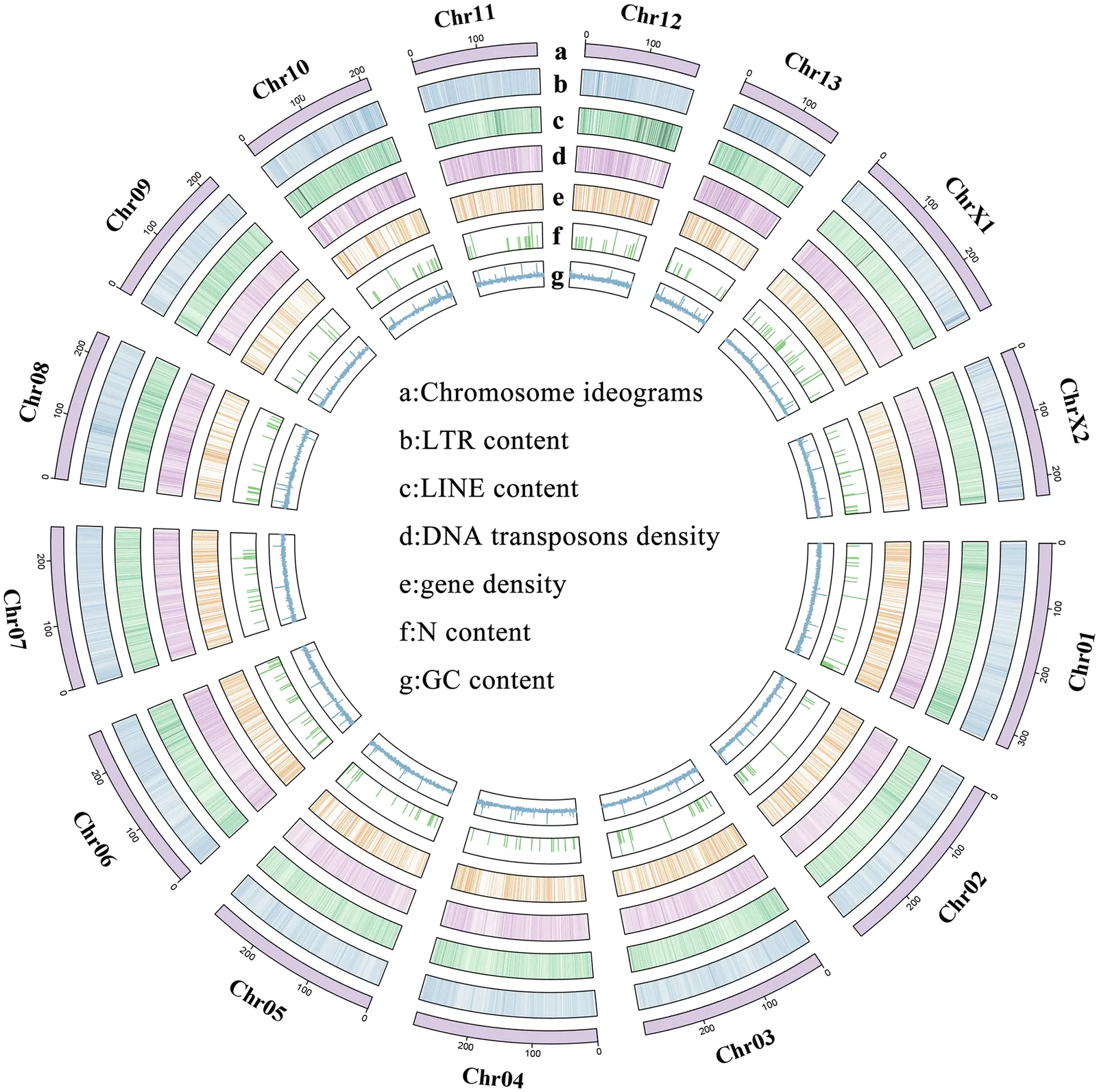
Genomic features of *Spartaeus platnicki*. See Tables 2–4 for more detailed statistics.

Building on the first reference genome of Salticinae, *Salticus scenicus* (BioProject: PRJEB72162), generated by the Wellcome Sanger Institute Tree of Life Programme, we present the first chromosome-level genome assembly for a basal lineage jumping spider. Genome sequencing of *S. platnicki* yielded 742.97 Gb of clean data from PacBio HiFi, Illumina, Hi-C, and RNA-seq platforms (Table 1). The final assembly spans 3.71 Gb, with a scaffold N50 of 262 Mb, comprising 15 pseudo-chromosomes and 97 unplaced scaffolds (Fig. 2, Table 2). The assembly exhibits high completeness, with BUSCO score of 98.60% (Table 2). Repetitive elements account for 65.57% (2.44 Gb) of the genome (Table 3). A total of 15,660 protein-coding genes were predicted (Table 2), with a BUSCO completeness score of 97.60%. This high-quality, chromosome-level assembly and comprehensive annotation provide a valuable genomic resource. It fills a fundamental phylogenetic gap in spider genomics and provides a cornerstone for comparative evolutionary studies across the entire Salticidae family.

**Table 1:** Statistics of the DNA sequence data used for the *de novo* genome assembly of *Spartaeus platnicki*.

Type	Number of reads	Data size (Gb)	Length per read (bp)	Length N50 (bp)	Sequencing coverage (×)
WGS	2,358,701,388	353.81	150	—	95.26
HiFi	8,664,664	122.80	14,172.11	14,070	33.06
Hi-C	1,589,678,130	238.45	150	—	64.20
RNA-sr	111,089,494	16.66	150	—	—
RNA-ONT	5,083,839	11.25	2,212.58	2,615	—

**Table 2:** Summary statistics of the *Spartaeus platnicki* genome assembly and annotation.

Assembly	Content	Statistics
	Assembly size (bp)	3,714,248,756
	Number of pseudo-chromosomes (sizes bp)	15 (3,690,651,725)
	Number of scaffolds/contigs	97/349
	Longest scaffold/contig (Mb)	323.776/127.383
	N50 scaffold/contig length (Mb)	262.304/26.018
	GC content (%)	33.87
	BUSCO completeness (%)	98.60
	S	93.60
	D	5.00
	F	0.20
	M	1.20
**Mapping ratio of reads (%)**	BGI	98.07
	HiFi	99.91
	RNA-sr	90.30
	RNA-ONT	98.97
**Structure Annotation**	**Protein-coding genes**	
	Number of protein-coding genes	15,660
	Number of predicted protein sequences	19,504
	Mean gene length (bp)	69,188.10
	Mean protein length (aa)	564.30
	BUSCO completeness (%)	97.60
	S	74
	D	23.6
	F	0.1
	M	2.3
	**Exon**	
	Mean number of exons per gene	8.80
	Mean exon length (bp)	354.80
	**CDS**	
	Mean number of CDSs per gene	8.40
	Mean CDS length (bp)	195.80
	**Intron**	
	Mean number of introns per gene	7.70
	Mean intron length (bp)	8,829.40
**Function Annotation**	**Type**	**Number**
	UniProt	14,909 (95.20%)
	InterProScan	12,964 (82.78%)
	eggNOG	14,007 (89.44%)
	KEGG orthologs (ko)	9,459 (60.40%)
	KEGG pathway	5,917 (37.78%)
	GO (combined)	11,784 (75.24%)

**Table 3:** Summary of repetitive elements in *Spartaeus platnicki*.

Type	Number	Length (bp)	Percentage (%)
DNA	2,683,855	812,690,195	21.88
LINE	615,705	192,459,906	5.18
LTR	1,116,487	329,456,451	8.86
SINE	226,012	43,916,157	1.18
Unknown	5,122,020	1,057,366,351	28.47
Total	9,764,079	2,435,889,060	65.57

## Methods

### Sample collection, extraction, and sequencing

Multiple adult females and males of *Spartaeus platnicki* (NCBI: txid426839) were collected from Huaping National Nature Reserve, Guangxi Zhuang Autonomous Region (GZAR), China in 2024. The fresh samples were immediately flash-frozen in liquid nitrogen and then stored at −80 °C until use. The species identification was conducted through a meticulous comparison of somatic and genitalic characteristics of the specimens with reliable and authoritative taxonomic literature. Ethical approval for animal use was not required for this study.

A multi-platform sequencing strategy was employed to generate a high-quality, chromosome-level reference genome for *S. platnicki*. Genomic DNA was extracted from fresh tissue using the cetyltrimethylammonium bromide (CTAB) method. DNA integrity was assessed by agarose gel electrophoresis, and purity was evaluated using spectrophotometric measurements to meet the standards required for library construction. To achieve both high accuracy and long contiguity, short-read and long-read sequencing technologies were combined.

Short-insert (200–400 bp) paired-end libraries were prepared for both female and male individuals using the Agencourt AMPure XP-Medium Kit (Beckman Coulter, USA) and sequenced on the DNBSEQ-T7 platform. The short-read data were primarily used for *k-mer* analysis to estimate the genome size, heterozygosity, and repeat elements, as well as for assembly polishing and annotation support. In addition, sex chromosomes were inferred by comparing the sequencing depth difference across the assembled scaffolds in the male sample. Long-read sequencing was conducted using the PacBio Sequel Revio platform. Genomic DNA was sheared into ~20 kb fragments using the Megaruptor (Diagenode B06010001, Liege, Belgium) and concentrated with AMPure® PB Beads (Pacific Biosciences 100-265-900). Libraries were constructed with the SMRTbell® Express Template Prep Kit 3.0 (Pacific Biosciences #PN 101-853-100, CA, USA), including overhang removal, DNA damage repair, end repair, A-tailing, adapter ligation, and nuclease digestion. Size selection was performed using the SageELF (Sage Science ELF000) or BluePippin (Sage Science BLU0001) systems to obtain uniform 20 kb inserts. All PacBio HiFi sequencing was conducted by Berry Genomics (Beijing, China).

To achieve chromosome-level scaffolding, Hi-C sequencing was performed to capture the three-dimensional chromatin conformation. Chromatin was cross-linked with formaldehyde, digested with MboI, end-repaired, biotinylated, proximity-ligated, de-crosslinked, and purified for Hi-C library construction. Hi-C libraries were sequenced on the DNBSEQ-T7 platform by Berry Genomics.

For transcriptome sequencing, total RNA was extracted using the TRIzol™ Reagent Kit (Invitrogen, USA), and strand-specific mRNA-seq libraries were prepared with the VAHTS mRNA-seq v2 Library Prep Kit (Vazyme, China) to support gene structure prediction and functional annotation. To obtain full-length transcripts for precise gene model annotation, RNA was extracted using the RNAprep Pure Plant Plus Kit (DP441), and ONT PromethION libraries were prepared using SQK-PCS109 and SQK-PBK004 kits (Benagen, Wuhan, China).

All sequencing data were integrated through a hybrid assembly workflow, in which PacBio HiFi reads served as the assembly backbone, Illumina reads were used for polishing, and Hi-C data provided chromosome anchoring. Transcriptomic data—including both short-read RNA-seq and full-length ONT transcripts—were incorporated to assist gene prediction and functional annotation, ultimately yielding a chromosome-level, high-quality reference genome.

### Genome survey and assembly

Quality control and trimming of the raw BGI next-generation sequencing data were performed using fastp v0.23.4^6^. Specifically, bases with quality scores below 20 were removed (>Q20), duplicate sequences were discarded (-D), poly-G/X tails were trimmed (-g -x), reads containing more than 10% low-quality bases were filtered out (-u 10), and base correction was conducted using overlapping reads (-c). Genome survey was conducted based on the *k-mer* profiling, with *k-mers* counted using khist.sh (BBMap v38.90^7^). Using a *k-mer* size of 21, the haploid genome size estimated to be 3.63 Gb, with a heterozygosity of 1.46% and a repeat sequence of 58.91%.

HiFi reads were generated using pbccs v6.4.0^8^ with a minimum base quality of Q20. *k-mer* spectra (21-mer and 31-mer) were constructed using yak v0.1^9^ for downstream analysis. *De novo* assembly of the HiFi reads was performed using Hifiasm v0.24.0^10^ with the ‘-l 3’ parameter, which removes contigs with coverage below 3 × (1/10 of the mean) to minimize artifacts from sequencing errors or contamination. The assembly was polished iteratively with NextPolish2 v0.2.1^11^: the first round used HiFi reads alignments generated by Minimap2 v2.7 (-ax lr:hqae)^12^, followed by two rounds with Illumina paired-end reads. Redundant contigs were subsequently removed with Purge_dups v1.2.5^13^ based on sequence similarity and coverage depth using default settings (-2 -a 70).

**Fig. 3:**
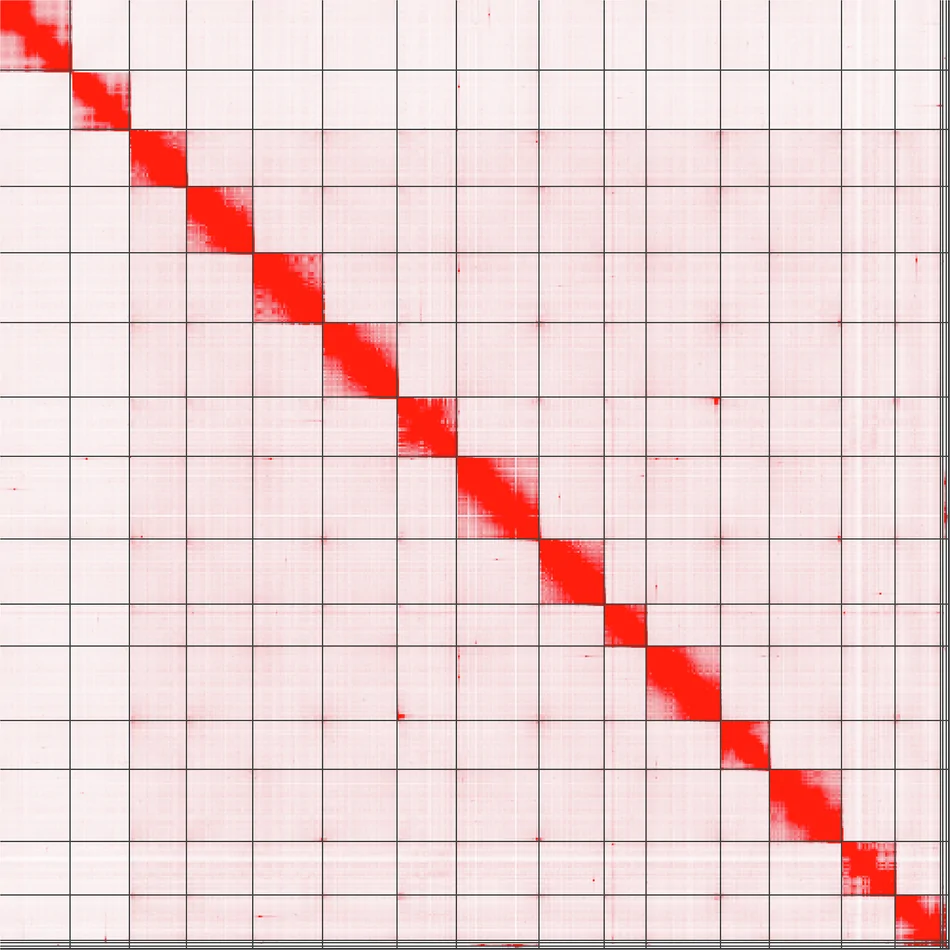
Hi-C interaction heatmap of the 15 pseudo-chromosomes in *Spartaeus platnicki*. Darker color indicates higher interaction frequencies.

The chromosome-level assembly was generated by scaffolding contigs into pseudo-chromosomes using Hi-C data. Hi-C reads were processed with chromap v0.2.6^14^ for alignment, duplicate removal and extraction of valid interaction pairs. Scaffolding was carried out in two rounds with YAHS v1.2^15^ under default parameters. Following the first round, the assembly was inspected and manually corrected with Juicebox v1.11.08^16^ based on Hi-C contact heatmap patterns, and the corrected assembly was then used as input for a second round of scaffolding with YAHS to produce the final chromosome-scale pseudo-chromosomes (Fig. 3).

The coverage of pseudo-chromosomes was estimated using SAMtools v1.10^17^, with input alignment files generated by Minimap2 from HiFi and WGS reads. Assembly completeness was assessed with BUSCO v5.7.1^18^ against the arachnida_odb10 database. In addition, WGS and RNA-seq data were aligned back to the assembled genome using Minimap2 to evaluate assembly completeness and accuracy, and alignment rates were calculated using SAMtools. Potential contamination in the assembly was screened with MMseqs2 v13^19^, using BLASTN-like searches against the NCBI nucleotide (nt) database and the UniVec Core database. Assembly accuracy was evaluated with Merqury v1.3^20^, which estimates the consensus quality value (QV) and produces *k-mer* spectrum plots based on *k-mer* co-occurrence between the assembly and HiFi sequencing reads. The final genome assembly was 3.71 Gb with 99.36% of the sequences assembled into 15 pseudo-chromosomes (Fig. 2, Table 2).

### Transposable element annotation

A *de novo* repeat library was initially constructed using RepeatModeler v2.0.5^21^ with LTR structure detection enabled (-LTRStruct). This library was subsequently merged with Dfam 3.7^22^ and RepBase-20181026^23^ to generate the final reference repeat database. Repetitive elements were identified and masked using RepeatMasker v4.1.5^24^ against the reference repeat library. In total, repeats spanned 2.44 Gb, accounting for 65.57% of the genome (Table 3). The most abundant repeat types were unclassified elements (28.47%), followed by DNA transposons (21.88%), LTR retrotransposons (8.86%), LINEs (5.18%) and SINEs (1.18%).

**Fig. 4:**
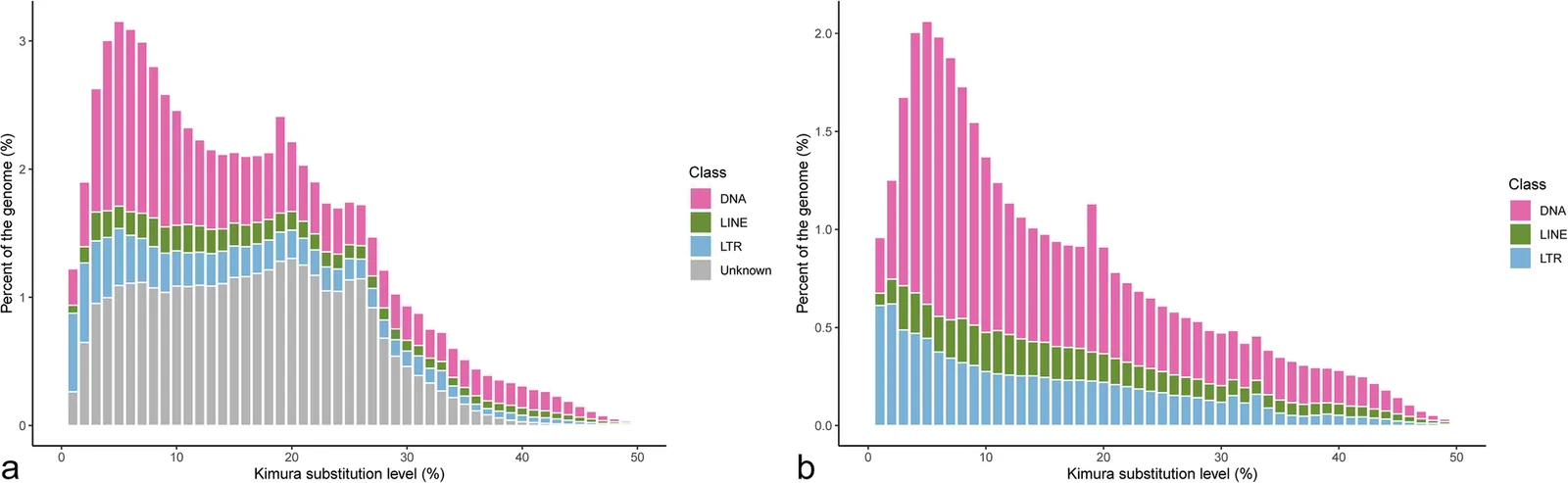
Interspersed repeat landscape based on CpG-adjusted Kimura 2-parameter (K2P) divergence. (**a**) Including unclassified repeats; (**b**) Excluding unclassified repeats.

To further investigate the expansion of transposable element (TE), the TE landscape was assessed using the calcDivergenceFromAlign.pl script included in RepeatMasker v4.1.5, which calculates the Kimura 2-Parameter (K2P) divergence for each TE family. Our findings suggest that the TEs of *Spartaeus platnicki* may have undergone three rounds of expansion (Fig. 4).

### Non-coding RNA annotation

Non-coding RNAs (ncRNAs) were annotated by identifying rRNAs, snRNAs and miRNAs though searches against the Rfam database using Infernal v1.1.5^25^, and by predicting tRNAs with tRNAscan-SE v2.0.12^26^. Low-confidence tRNAs were filtered out using “EukHighConfidenceFilter” script provided in tRNAscan-SE. A total of 14,600 ncRNAs were predicted in the genome (Table 4).

**Table 4:** Summary of non-coding RNAs in *Spartaeus platnicki*.

Class	Copies
riboswitch	1
ribozyme	2,015
miRNA	4,098
leader	1
rRNA	116
sRNA	2
snRNA	775
tRNA	7,034
Other	558
Total	14,600

### Protein-coding gene annotation

Protein-coding genes were predicted from the repeat-masked genome using MAKER v3.01.04^27^, following an integrative strategy that combined *ab initio* prediction, transcript evidence, and protein homology support.

For *ab initio* gene prediction, BRAKER v3.0.6^28^ and GeMoMa v1.9^29^ were employed, integrating RNA-seq and protein evidence to improve accuracy. BRAKER performed automated training of Augustus v3.4.0^30^ and GeneMark-ETP^31^, incorporating arthropod protein sequences from OrthoDB11^32^ and transcriptome alignments. GeMoMa, which leverages protein homology and intron position conservation, was run with parameters “GeMoMa.c = 0.4 GeMoMa.m = 200000” using high-quality genome assemblies, protein sequences, and annotation files from one insect (*Drosophila melanogaster*), one crustacean (*Daphnia magna*), and four arachnids (*Dermacentor silvarum*, *Tetranychus urticae*, *Argiope bruennichi*, and *Uloborus diversus*). Predictions from BRAKER and GeMoMa were merged into a single file (ab.gff3) and supplied to MAKER as *ab initio* evidence.

Transcript-based evidence was obtained by aligning RNA-seq reads to the genome with HISAT2 v2.2.1^33^, followed by genome-guided transcript assembly using StringTie v2.2.1^34^. Homology-based evidence was incorporated using the same protein dataset applied in the GeMoMa analysis.

The final structural annotation predicted 15,660 protein-coding genes, with an average of 8.80 exons per gene, mean exon length of 354.80 bp, and mean intron length of 8,829.40 bp (Table 2).

Predicted proteins were first searched against protein databases, including UniProtKB (Swiss-Prot + TrEMBL), using Diamond v2.1.7.161^35^ in very sensitive mode (–very-sensitive -e 1e-5) to assign putative gene functions. Conserved domains, motifs, Gene Ontology (GO) terms, and pathway information (KEGG, Reactome) were further annotated by searching the Pfam^36^ database with InterProScan v5.70–102.0^37^ and by using eggNOG-mapper v2.1.12^38^ against the eggNOG v5.0.2^39^ database. The results from these analyses were integrated to generate the final functional annotation of protein-coding genes (Table 2).

### Visualization of genomic features

Genomic feature analysis was conducted using TBtools II v2.363^40^. Chromosome length, the proportion of unknown bases (N), and GC content were calculated with the *“Fasta Stats”* module. The spatial distributions of repetitive elements (including LTRs, LINEs, and DNA transposons) and gene density were analyzed in non-overlapping 100-kb windows based on repeat and gene annotation files. All datasets were normalized and integrated in TBtools II to generate a Circos plot (Fig. 2) for visualization.

## Data Records

All raw sequencing data have been deposited in the NCBI Sequence Read Archive (SRA) under the accession numbers SRR31847034–SRR31847037^41–44^ for the female and SRR31842152–SRR31842154^45–47^ for the male. The genome assembly has been released under JBHFNZ000000000^48^. The annotation files are available in Science Data Bank^49^.

## Technical Validation

Potential contaminant sequences were detected using BLASTN-like searches against the NCBI nucleotide (nt) and UniVec databases with a minimum sequence identity threshold of 0.8 (‘–min-seq-id 0.8’) implemented in MMseqs2 v13. Sequences showing over 80% similarity were further examined through online BLASTN searches against the NCBI nt database, and those exhibiting more than 90% identity were considered contaminants and removed from the assembled scaffolds.

The completeness of assembly was assessed using BUSCO v5.7.1 with the arthropoda_odb 10 dataset (n = 2,934). Mapping rates were estimated by aligning the clean reads of Illumina and PacBio sequences to the final assembly using Minimap2. The assembled genome showed a BUSCO completeness of 98.60% (Table 2).

Based on BUSCO evaluation, the predicted protein-coding genes were 74.0% single-copy, 23.6% duplicated, 0.1% fragmented, and 2.3% missing orthologs (Table 2).

## Data Availability

All data processing was performed strictly following the instructions and guidelines of the respective bioinformatic software packages, and no custom scripts or in-house code were used in this study.
